# l-Arginine Improves Solubility and ANTI SARS-CoV-2 Mpro Activity of Rutin but Not the Antiviral Activity in Cells

**DOI:** 10.3390/molecules26196062

**Published:** 2021-10-07

**Authors:** Luca Sancineto, Carmine Ostacolo, David Ortega-Alarcon, Ana Jimenez-Alesanco, Laura Ceballos-Laita, Sonia Vega, Olga Abian, Adrian Velazquez-Campoy, Silvia Moretti, Agnieszka Dabrowska, Pawel Botwina, Aleksandra Synowiec, Anna Kula-Pacurar, Krzysztof Pyrc, Nunzio Iraci, Claudio Santi

**Affiliations:** 1 Group of Catalysis Synthesis and Organic Green Chemistry, Department of Pharmaceutical Sciences, University of Perugia, Via del Liceo 1, 06122 Perugia, Italy; luca.sancineto@unipg.it; 2 Department of Pharmacy, University of Naples Federico II, 80131 Napoli, Italy; carmine.ostacolo@unina.it; 3 Institute for Biocomputation and Physics of Complex Systems (BIFI), Joint Units IQFR-CSIC-BIFI and GBsC-CSIC-BIFI, Universidad de Zaragoza, 50018 Zaragoza, Spain; dortega@bifi.es (D.O.-A.); ajimenez@bifi.es (A.J.-A.); ceballos.laita@gmail.com (L.C.-L.); svega@bifi.es (S.V.); oabifra@unizar.es (O.A.); adrianvc@unizar.es (A.V.-C.); 4 Departamento de Bioquímica y Biología Molecular y Celular, Universidad de Zaragoza, 50009 Zaragoza, Spain; 5 Instituto de Investigación Sanitaria de Aragón (IIS Aragon), 50009 Zaragoza, Spain; 6 Instituto Aragonés de Ciencias de la Salud (IACS), 50009 Zaragoza, Spain; 7 Centro de Investigación Biomédica en Red en el Área Temática de Enfermedades Hepáticas Digestivas (CIBERehd), 28029 Madrid, Spain; 8 Fundación ARAID, Gobierno de Aragón, 50018 Zaragoza, Spain; 9 Eco Tech Engeneering e Servizi Ambientali srl, Via Bruno Colli 4, 06135 Ponte San Giovanni, Italy; silvia.moretti@ecotechgroup.it; 10 Malopolska Centre of Biotechnology, Virogenetics Laboratory of Virology, Jagiellonian University, Gronostajowa 7a, 30-387 Krakow, Poland; agnieszka.dabrowska@doctoral.uj.edu.pl (A.D.); pawel.botwina@doctoral.uj.edu.pl (P.B.); a.synowiec@doctoral.uj.edu.pl (A.S.); anna.kula-pacurar@uj.edu.pl (A.K.-P.); k.a.pyrc@uj.edu.pl (K.P.); 11 Microbiology Department, Faculty of Biochemistry, Biophysics and Biotechnology, Jagiellonian University, Gronostajowa 7, 30-387 Krakow, Poland; 12 Department of Chemical, Biological, Pharmaceutical and Environmental Sciences, University of Messina, Viale Ferdinando Stagno d’Alcontres 31, 98166 Messina, Italy

**Keywords:** rutin, l-arginine, SARS-CoV-2, main protease, quercetin

## Abstract

The COVID-19 pandemic outbreak prompts an urgent need for efficient therapeutics, and repurposing of known drugs has been extensively used in an attempt to get to anti-SARS-CoV-2 agents in the shortest possible time. The glycoside rutin shows manifold pharmacological activities and, despite its use being limited by its poor solubility in water, it is the active principle of many pharmaceutical preparations. We herein report our in silico and experimental investigations of rutin as a SARS-CoV-2 Mpro inhibitor and of its water solubility improvement obtained by mixing it with l-arginine. Tests of the rutin/l-arginine mixture in a cellular model of SARS-CoV-2 infection highlighted that the mixture still suffers from unfavorable pharmacokinetic properties, but nonetheless, the results of this study suggest that rutin might be a good starting point for hit optimization.

## 1. Introduction

**Figure 1:**
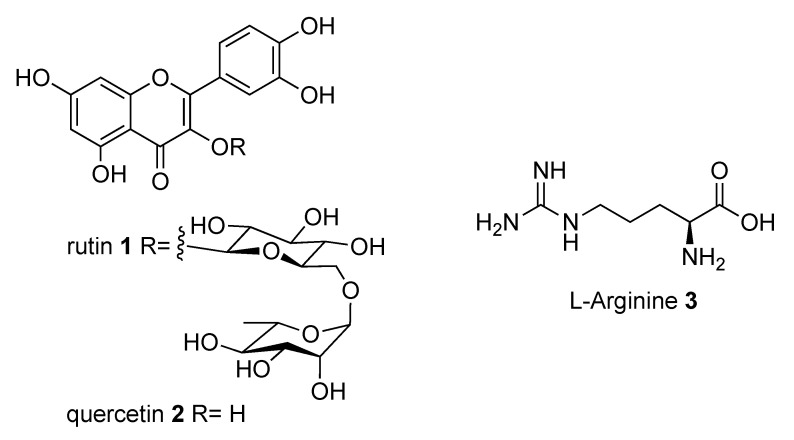
Structures of rutin (**1**), quercetin (**2**), and l-arginine (**3**).

The glycoside rutin (3′,4′,5,7-tetrahydroxyflavone-3-*O*-rutinoside) (compound **1**, Figure 1) is a widespread flavonoid found in many fruits and vegetables. It is extensively consumed in foods and folkloric medicine all over the world and has recently drawn the attention of the scientific community due to its manifold pharmacological activities [1]. Starting from its first isolation in early 1900 [2], several in vitro and in vivo activities were proven. Rutin has indeed antidiabetic [3], neuroprotective [4,5], antibacterial [6], and anti-inflammatory [7,8] properties, among others [9]. Besides, it showcases antioxidant activity [10] and lack of toxicity, and as a result, so far, more than 130 pharmaceutical preparations contain rutin as active principle.

Despite its commercial availability, the practical use of rutin is still problematic because of its unfavorable physicochemical properties. Rutin is indeed well soluble in ethanol, isopropyl alcohol, and other organic solvents, such as DMSO, but it is poorly water soluble (13 mg 100 cm^−3^) [11]. The lack of aqueous solubility strongly limits its application in pharmacology, as well as in cosmetology.

Many attempts have been carried out to solve such solubility issues. In particular, Savic and colleagues prepared inclusion complexes between rutin, β-cyclodextrin, and (2-hydroxypropyl)-β-cyclodextrin endowed with a better water solubility and antioxidant activity, as assessed by the DPPH assay. Additionally, the photostability profile of the inclusion complexes was superior when compared to that of rutin alone [11]. 

Particle size reduction is another method to improve rutin dissolution velocity and solubility [12], as well as its formulation as nanocrystals [13]. 

The DPPH radical scavenging activity and the water solubility of rutin were strikingly improved in the presence of cationic, anionic, non-ionic, and mixed aqueous micelles [14]. 

Very recently, some of us reported that quercetin (compound **2**, Figure 1), the flavonoid core of rutin, potently inhibited the activity of the SARS-CoV-2 main protease (Mpro), plausibly binding to its active site, with a Ki of 7.4 µM in the presence of NaCl [15]. In line with this, a series of quercetin analogues were prepared and tested for their ability to inhibit not only the enzyme but also the viral replication in a cellular context, finding some compounds endowed with a better pharmacological profile when compared to quercetin [16]. Moreover, some in silico studies proposed rutin, other flavonoids, and natural products as potential inhibitors of SARS-CoV-2 key proteins [17,18,19,20,21].

In the frame of our ongoing efforts toward the identification of suitable antimicrobial compounds [22,23,24,25], here we report the preparation, the anti Mpro evaluation, and the in silico studies of the rutin: l-arginine 1:2 mixture (hereafter named ***RutinArg***). This combination has been identified as a good water-soluble version of rutin following a study about the development of a green deuteration procedure of flavonoids [26]. In particular, NMR analysis evidenced that, when using less than two moles of **3** for each mole of **1,** the latter was not fully solubilized even after mild heating at 60 °C. The proton spectrum of this mixture recorded in D_2_O showed the two species in a ratio 2:1, respectively, without relevant changes in the chemical shifts of the parent compounds. Furthermore, l-arginine is endowed with antioxidant capacity and has already proved to enhance the pharmacological properties of rutin in vivo. [27] The solubility improvement of **1** in the presence of **3**, described in our precedent report [26], [from 0.013% (*w*/*v*) to 33% (*w*/*v*) of rutin in water] was confirmed also for ***RutinArg***.

Here we are reporting the protease inhibitory activity of ***RutinArg*** compared with that of the native rutin, l-arginine (**3**) and quercetin, and a theoretical investigation that explains the observed solubility and investigates the rutin/Mpro interaction.

We decided to focus our attention on rutin since, among the almost 180 different quercetin-containing glycosides in nature, it is one of the most common in vegetables, fruits, and beverages and represents the main dietary sources of quercetin [28]. Moreover, quercetin is commonly obtained from rutin by catalyzed hydrolysis [29].

## 2. Results and Discussion

**Figure 2:**
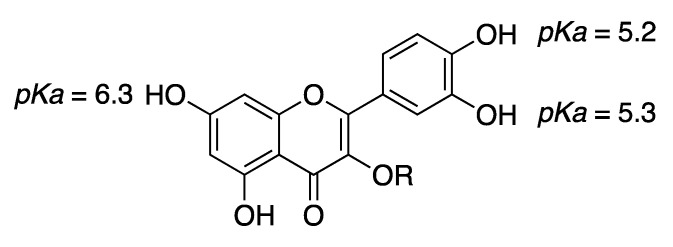
Jaguar-predicted *pKa* values for the three acidic hydroxyl groups of the quercetin moiety. R represents the rutin glycon.

***RutinArg*** was prepared by grinding in a mortar a 1:2 mixture of rutin (**1**) and l-arginine (**3**) to give a mixture that can be fully solubilized in water by heating at 80 °C for 1 min, thus affording a solution containing 14 mg/mL of rutin that has been used for NMR analysis. Three hydroxyl groups of rutin, i.e., those in positions 7, 3′ and 4′ of the aglycon, possess acidic character. In particular, *pKa* calculations by means of the Jaguar program [30,31,32] estimated a *pKa* values of 6.3 for the hydroxyl group at C7 and of 5.2 and 5.3 for the hydroxyl groups at 3′ and 4′, respectively. The basic character of **3** might promote the ionization of the above-mentioned hydroxyl groups, thus increasing its solubility (Figure 2).

We simulated the neutral (i1) and the three ionized states i2-4 of rutin by replica exchange with solute tempering (REST) molecular dynamics (MD) [33,34] to estimate the interaction energies of rutin with water and Na^+^ and Cl^−^ ions. As expected, the interaction of rutin with the aqueous environment is predicted to be much more favorable when rutin is ionized, thus explaining the improved solubility of rutin in the arginine-alkalinized aqueous environment (Table 1).

**Figure 3:**
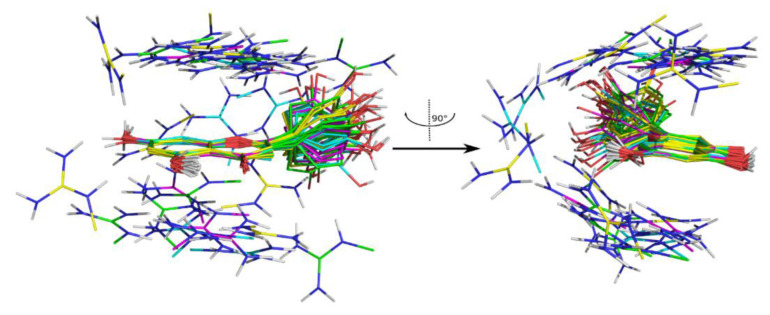
REST MD trajectories clustering of i1-4 rutin/l-arginine. The structure nearest to the centroid for each highly populated cluster (>100 members) is represented in sticks and colored according to the following scheme: i1—magenta, i2—yellow, i3—green, i4—cyan.

Another series of REST MD simulations was run adding l-arginine to the simulated systems to get insight into possible specific interactions between l-arginine and i1-i4 rutin. Indeed, cluster analyses of the MD trajectories highlight that the guanidine group of the amino acid is often found in a π-cation stacking interaction with the quercetin rings. In particular, this interaction is found in 2899 out of 10,000 trajectory frames for i1, in 2908 out of 10,000 for i2, in 2285 out of 10,000 for i3, and in 2622 out of 10,000 for i4. Figure 3 depicts, for each highly populated cluster (>100 members), the structure nearest to the cluster centroid.

Such π-cation stacking interactions may hamper the formation of the π-π stacking interaction observed in crystal structures of rutin [35], as well as of other related flavonoids [36], thus contributing to the improved aqueous solubility of the ***RutinArg*** mixture. It is worth noting that l-arginine-mediated solubility improvement involves chemical features that are located on the aglycon. It is thus plausible that a similar solubility improvement could be observed in other quercetin derivatives.

***RutinArg*** was assayed together with the nude **1** and quercetin (**2**), through the in vitro assay based on Mpro hydrolytic activity using a Förster resonance energy transfer (FRET) substrate, which was very recently implemented by some of us [15,37]. Quercetin (**2**) was selected as comparator because it proved to efficiently inhibit the viral enzyme. First, all of the compounds were tested at the fixed concentration of 100 μM, progressing in further testings those compounds capable of completely abolishing enzyme activity. (Results are collected in Table 2).

**Figure 4:**
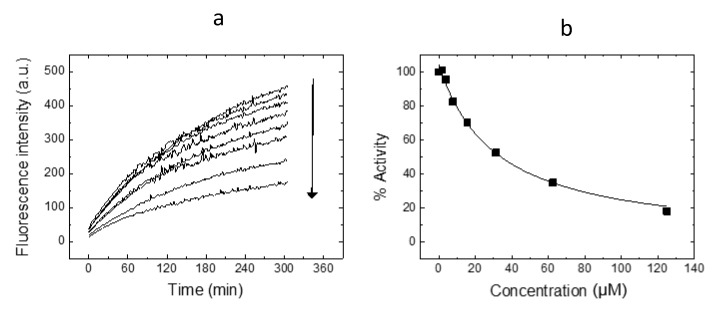
Inhibition curve for ***RutinArg*** against Mpro. (**a**) Time evolution of the substrate fluorescence as a function of time. The observed FRET diminishes as the substrate is hydrolyzed by Mpro, reflecting the spatial separation of the donor-acceptor FRET couple. As the ***RutinArg*** concentration increases (arrow), the activity of Mpro, quantitated as the initial slope of each trace, decreases. (**b**) Inhibition curve obtained by plotting the activity percentage of Mpro, calculated as the ratio of the initial slope at each ***RutinArg*** concentration by that corresponding to no compound added (control), as a function of the ***RutinArg*** concentration. The non-linear least-squares analysis of the data considering a ligand-depletion model provided an estimation of the inhibition constant K_i_. When the enzyme concentration and the ligand depletion are not accounted for, the effective inhibition concentration IC_50_ is estimated.

Interestingly, while arginine **3** was devoid of any anti-Mpro activity, the ***RutinArg*** mixture significantly inhibited the enzyme at 100 µM concentration. Subsequently, it was tested at serial concentrations in order to establish the IC_50_ and the K_i_ values. As shown in Figure 4, ***RutinArg*** showed a dose-dependent inhibition activity against Mpro with an estimated K_i_ of 11 µM. This value is similar to that displayed by compound **2**, thus indicating that the sugar moiety might provide a minor contribution to the interaction with the enzyme-active site. Notably, the small difference in K_i_ for rutin and ***RutinArg*** is statistically significant since it is larger than the experimental error (Figure 4). Inhibition assays with arginine showed that Mpro was 100% active in any arginine concentration tested. Therefore, a K_i_ or IC_50_ could not be estimated for this compound.

The interactions between rutin and SARS-CoV-2 Mpro, were studied in silico by molecular modeling, highlighting contacts of the aglycon with the catalytic dyad and a substantial lack of interaction with the enzyme by the terminal sugar moiety, in partial accordance with a previous report [37]. Briefly, we docked i1-4 rutin into Mpro by means of Glide SP, obtaining five distinct bound conformations for each rutin ionization state. In turn, the bound conformations were rescored in terms of the free energy of binding using the Prime-MMGBSA program. The best scoring bound conformation (Prime-predicted ΔG_bind_ = −71.73 kcal/mol) was then submitted for molecular dynamics simulation to inspect the rutin/Mpro interactions.

**Figure 5:**
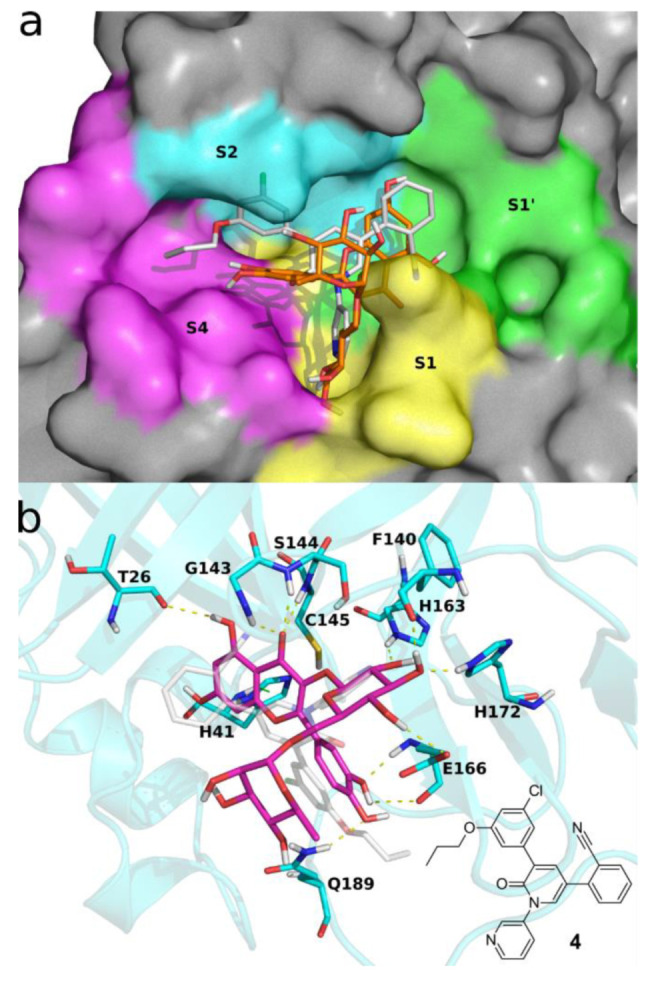
Docking pose of **1** into SARS-CoV-2 Mpro active site. (**a**) **1** is shown in orange sticks; the molecular surface of Mpro is colored according to the binding sites S1 (yellow), S1′ (green), S2 (cyan) and S4 (magenta). (**b**) **1** is represented in magenta sticks and SARS-CoV-2 Mpro in cyan cartoons and sticks; dashed lines represent h-bonds (in yellow) and π-π stacking interactions (in green). Experimental bound conformation of **4** (PDB 7L11) [40] is shown in white sticks for reference in both panels. These figures were made using open source PyMOL v. 1.8.4.0.

As shown in Figure 5 panel a, rutin docks into Mpro active site, binding to S1, S1′, and S4 sites residues. (For a description of the sites and the role of the main residues they are made up of, see ref [38].)

**Figure 6:**
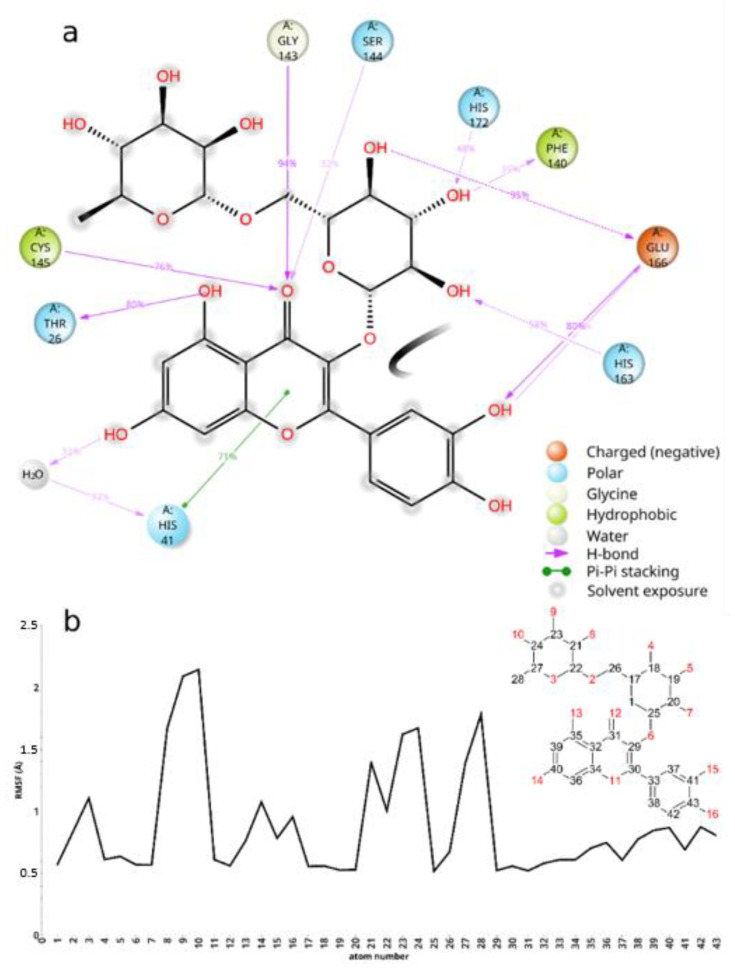
Results summary of rutin/Mpro MD simulations. (**a**) Rutin/Mpro interaction diagram. Only interaction detected for at least 36 ns out of 120 ns of simulation time are shown. (**b**) Root mean square fluctuations of rutin atoms during 120 ns of MD simulation.

The binding of rutin to Mpro is stabilized by an intricate network of interaction with several residues of the active site (Figure 5a,b and Figure 6a). The fused rings of the flavone dock into S1′, creating as well some interaction with S1 residues, such as Ser144 and Cys145. The carbonyl oxygen might get involved in three hydrogen bonds with Gly143, Cys145, and Ser 144. The hydroxyl at the aglycon C5 makes a hydrogen bond with Thr26, while the one at C7 makes a water-mediated hydrogen bond with His41. The latter residue, which together with Cys145 forms the Mpro catalytic dyad, stabilizes rutin binding by π-π stacking with the aglycon fused ring system. Other in silico studies used different methodologies, force-fields, and target structures and predict the aglycon phenyl moiety docking in close proximity to S1′ [17] or S1 [37]; in contrast, in our model the phenyl moiety docks into S4, making H-bonds with the backbone of Glu166, while the glucopyranose moiety docks into S1, making H-bonds with Phe140, His163, Glu166, and His 172. The rhamnopyranosyl moiety does not make any stable interaction during the MD simulation, and its root mean square fluctuations are the highest among rutin atoms (Figure 6b). Thus, while the aglycon is found to occupy the catalytic center and interact with the catalytic dyad, the glucopyranose might interact with residues His163, His172, and Glu166, that are believed to provide the opening gate for the substrate in the active state of the protomer [39]. The information gathered from these simulations might be accounted for when attempting to improve the activity of quercetin derivatives.

Finally, to test the ability of rutin **1** and its mixture with arginine **3** to inhibit the SARS-CoV-2 replication in a cellular model, a preliminary cytotoxicity experiment was carried out. In particular, compounds **1**, **3** and ***RutinArg*** were tested at the concentrations of 1000–500–100–50–10 µM in A549^ACE2+/TMPRSS2+^ cells without showing any signs of cytotoxicity at all tested doses, indicating that the compounds are safe for the cell in which the viral infection is mimicked. Interestingly, while ***RutinArg*** was fully soluble in the assay buffer and thus tested as it was, compound **1** needed to be dissolved first in DMSO and then diluted with the buffer. 

Disappointingly, neither compound **1** and **3** nor the ***RutinArg*** (dissolved in DMSO or water) were able to block viral replication when tested in the same cell line where the cytotoxicity was assayed, even at the highest tested concentrations (see Figure S1 in Supplementary materials). This indicates that, in this specific cell line, the compounds are devoid of antiviral activity. Most probably, the unfavorable pharmacokinetic profile plays a role in both the lack of activity and cytotoxicity.

## 3. Materials and Methods

Solvents and reagents are commercially available and were used as received. NMR spectroscopic experiments were obtained at 25 °C on Bruker DRX spectrometers operating at 400 MHz for the proton (Bruker, Fällanden, Switzerland). ^1^H and ^13^C chemical shifts (δ) are reported in parts per million (ppm), and they are relative to TMS 0.0 ppm. Data are reported as follows: chemical shift (multiplicity, coupling constants, where applicable, and the number of hydrogens). Abbreviations are as follows: s (singlet), d (doublet), t (triplet), q (quartet), dd (doublet of doublet), dt (double of triplet), tt (triplet of triplet), m (multiplet), br.s. (broad signal). Coupling constant (*J*) quoted in hertz (Hz) to the nearest 0.1 Hz.

Preparation of ***RutinArg***: 5.7 g (32 mmoles) of Arginine (Sigma-Aldrich, St. Louis, MO, USA) and 10 g (16 mmoles) of rutin (ACEF) were ground in a mortar for ten minutes. The resulting pale yellow solid mixture can be stored at room temperature without any precautions, and no decompositions were observed after several days (up to 6 months).

^1^H-NMR (400 MHz, D_2_O): δ 7.30 (d, *J* = 2.3 Hz, 1H, H2′); 7.21 (dd, *J* = 8.5 and 2.3 Hz, 1H; H6′); 6.53 (d, *J* = 8.5 Hz, 1H, H5′); 5.92 (s, 0.35 H *, H6); 5.77 (s, 0.19 H *, H8); 4,58 (d, *J* = 7.7 Hz, 1H, sugars); 4.36 (s, 1H, sugars); 3.57 (d, *J* = 11.0 Hz, 1H, sugars); 3.5–3.47 (m, 1H, sugars); 3.43–3.33 (m, 4H, sugars); 3.28–3.07 (m, 6H, sugars plus 2H Arg); 2.97 (t, *J* = 6.8 Hz, 4H, Arg); 1.68–1.55 (m, 4H Arg); 1.5–1.35 (m, 4H, Arg); 0.92 (d, *J* = 6 Hz, 3H, CH_3_) ppm. ^13^CNMR (100 MHz, D_2_O): 177.31 (Arg); 176.58; 174.52; 159.41; 157.61 (2C); 156.70 (Arg); 154.04; 145.87; 132.77; 122.91; 118.38; 116.28; 115.28; 103.17; 102.08; 101.27; 100.97; 96.88; 75.75; 74.87; 73.66; 71.89; 70.13 (2C); 69.68; 68.73; 67.59; 54.75 (Arg); 40.69 (Arg); 28.93 (Arg); 24.10 (Arg); 16.51 ppm.

H * exchanged with D of D_2_O

Molecular Modeling. Rutin and l-arginine were sketched using the Schrodinger Maestro GUI [41] and were submitted to a conformational search in implicit water using the Mixed torsional/Low-mode sampling method implemented in MacroModel [42]. Torsion sampling was set to extended; 1000 steps per rotatable bond were used and the maximum number of steps was set to 10,000. Conformers were considered redundant when they had less than 0.5 Å as maximum atom deviation. Probability of torsion/rotation/translation was set to 0.5, and minimum and maximum low-mode move distances were set to 3.0 and 6.0, respectively. Global minima only were retrieved and used for the subsequent calculations. Rutin atoms *pK_a_* values were calculated using the Jaguar *pK_a_* module [30,31,32]. 

To account for the protonation state influence on molecule conformation, a thorough accuracy conformational search was performed on each species, and *pK_a_* calculations were done on a maximum of 10 conformers in a 6 kcal/mol energy window. A geometry optimization step was carried out in water, and SCF convergence settings were left to their default values. 

Automated molecular docking of rutin into Mpro active site was carried out using Glide [43,44,45]. Docking target structure (PDB 7L11) [40] was prepared as previously described [46,47], using the Schrodinger protein preparation utility [48]. Docking space was centered on the native ligand of 7L11 and defined as a 25 Å^3^ cubic box, constraining the ligand diameter midpoint into a nested 20 Å^3^ cubic box. Ligand nonpolar atoms (charge < 0.15) vdW radii were scaled by a 0.80 factor. The five best scoring bound conformations for each ionization state of rutin (i1-4) were retrieved, discarding as duplicates poses that showed both rms deviation less than 1.5 Å and maximum atomic displacement less than 2.0 Å. A total of twenty rutin/Mpro complexes (five for each rutin ionization state) were then submitted to Prime MM-GBSA [49,50] calculations to estimate their Δg_bind_. Minimization was used as sampling method, treating all residues within 5 Å from ligand atoms as flexible; OPLS4 [51] was used as force field. The complex with the best MM-GBSA-predicted Δg_bind_ was used for the following MD simulations.

MD simulations of i1-4 rutin with and without l-arginine and of the rutin/Mpro complex were set up and run using Desmond [52]. 

The simulated environments were built using the system builder utility, and solvation was treated explicitly using the TIP3P [53] water model in periodic boundary conditions cubic boxes. The systems were neutralized by Na^+^ and Cl^−^ ions, which were added until a 0.15 M concentration was reached. Before running the MD production stages, all systems were relaxed by a previously reported multi-stage protocol [54].

The neutral (i1) and the three ionized states i2-4 of rutin, with or without l-arginine, were simulated by replica exchange with solute tempering molecular dynamics to enhance the conformational sampling [33,34]. For each system, three replicas were run, setting the solutes as hot regions. To keep a replica exchange average acceptance ratio of around 0.3, the temperatures were set to 300–345–395.61 K and 300–337.5–379.68 K in the presence and absence of l-arginine, respectively.

Each replica was run for 1.2 µs in the NPT ensemble; pressure was set to 1.01325; and time steps were set to 2 fs, 2 fs, and 6 fs for bonded, near, and far interactions, respectively. Cutoff radius for short range interactions was set to 9 Å. MD simulation (120 ns) of the rutin/Mpro complex was run as previously reported [38].

MD simulations were analyzed using the Simulation Event Analysis, the Simulation Interactions Diagram, and the Conformer Cluster utilities of the Schrodinger Suite release 2021-1.

### 3.1. SARS-CoV-2 Mpro Expression and Purification

Mpro was expressed and purified as previously reported [15,16,37]. Briefly, Mpro was expressed in *E. coli* under isopropyl 1-thio-β-d-galactopyranoside (IPTG) induction at 18 °C for 5 h. Affinity chromatography using a cobalt column allowed fast purification in a single chromatographic step. Protein concentration was quantitated using an extinction coefficient of 32,890 M^−1^ cm^−1^ at 280 nm.

### 3.2. SARS-CoV-2 Mpro Proteolytic Activity Assay

A continuous assay based on Förster resonance energy transfer (FRET) to measure in vitro the catalytic activity of Mpro was implemented by using the substrate (Dabcyl) KTSAVLQSGFRKME (Edans)-NH2 (Biosyntan GmbH, Berlin, Germany) [55] [REF]. The enzymatic reaction was initiated by adding substrate (20 μM) to the enzyme (0.2 μM) in 100 μL volume. The reaction buffer was sodium phosphate 50 mM, pH 7, NaCl 150 mM, and DMSO 2.5%. Fluorescence emission was measured in a FluoDia T70 microplate reader (Photon Technology International, Birmingham, NJ, USA) for 20 min (excitation wavelength: 380 nm; emission wavelength: 500 nm). The initial slope of the time evolution curve of the fluorescence emission signal provided a direct quantification of the enzymatic activity. The Michaelis-Menten constant, *K*_m_, and the catalytic rate constant or turnover number, *k*_cat_, were previously estimated (*K*_m_ = 11 μM and *k*_cat_ = 0.040 s^−1^) [15].

### 3.3. SARS-CoV-2 Mpro Inhibition Assay

The in vitro inhibition potency of the compounds against Mpro was assessed through the estimation of the inhibition constant, K_i_, and the half-maximal inhibitory concentration, IC_50_, from experimental inhibition curves. Inhibition curves were obtained by measuring the hydrolytic activity of Mpro (at 0.2 μM) over substrate (20 μM) as a function of compound concentration (serial 2-fold dilution from 125 µM to 0 μM), maintaining the percentage of DMSO (2.5%) constant. The enzymatic activity, quantitated as the initial slope of the substrate fluorescence emission time evolution curve, was plotted as a function of compound concentration. The ratio between the activity (slope) in the presence and absence of the compound provides the residual percentage of activity at a given compound concentration. Non-linear regression analysis employing a simple inhibition model (considering inhibitor depletion due to enzyme binding) allowed us to estimate the inhibition constant, K_i_, and the effective concentration 50%, IC_50_ for each compound (if the finite concentration of enzyme and the ligand depletion due to enzyme binding are not explicitly accounted for in the model) for each compound [15,16].

### 3.4. Cells and Viruses

A549 cells are not susceptible to the SARS-CoV-2 infection, and therefore, they were modified to express the ACE2 receptor protein and the TMPRSS2 activating protease. Briefly, lentiviral vectors were prepared based on the pLKO.1-TRC-ACE2 (based on the Addgene plasmid # 10878) and pLEX307-TMPRSS2-blast (Addgene plasmid # 158458) plasmids. Wild-type A549 cells (ATCC CCL-185) were co-transduced with lentiviral vectors harboring TMPRSS2 and ACE2 genes. The medium was refreshed after 16 h, and cells were passaged 72 h post-transduction and fresh medium supplemented with puromycin (0.5 μg/mL, Bioshop, Burlington, ON, Canada) and blasticidin S (10 μg/mL, Sigma-Aldrich, St. Louis, MO, USA) for antibiotic selection to be applied. Cells were cultured for 2 weeks in the presence of puromycin and blasticidin S, and the clonal selection was performed afterward. Single cells were seeded in a 96-well plate and cultured for 3 weeks in 20% DMEM supplemented with puromycin (0.5 μg/mL) and blasticidin S (10 μg/mL). Single clones were analyzed by western blot, the clone with the highest ACE2, and TMPRSS2 expression was selected and propagated.

Vero (*Cercopithecus aethiops*; kidney epithelial; ATCC CCL-81) and A549 overexpressing ACE2 and TMPRSS2 (A549^ACE2/TMPRSS2^) cells were maintained in Dulbecco-modified Eagle’s medium (DMEM, high glucose, ThermoFisher Scientific, Warszawa, Poland) supplemented with 5% heat-inactivated fetal bovine serum (FBS, ThermoFisher Scientific, Poland). The medium was also supplemented with penicillin (100 U/mL, ThermoFisher Scientific, Warszawa, Poland) and streptomycin (100 μg/mL, ThermoFisher Scientific, Warszawa, Poland). Additional A549^ACE2/TMPRSS2^ cells were supplemented with blasticidin S (10 μg/mL, Sigma-Aldrich, St. Louis, MO, USA) and puromycin (0.5 μg/mL, Sigma-Aldrich, St. Louis, MO, USA) to maintain the only ACE2+TMPRSS2+ cells in the population. Cells were cultured at 37 °C in an atmosphere containing 5% CO_2_ and humidity. 

The SARS-CoV-2 strain used in the study was isolated in-house and is designated PL_P07 [GISAID Clade G, Pangolin lineage B.1] (accession numbers for the GISAID database: hCoV-19/Poland/PL_P07/2020). It was generated by infecting monolayers of Vero cells. The cells were incubated at 37 °C under 5% CO_2_. The virus-containing medium was collected at day 2 post-infection (p.i.), aliquoted, and stored at −80 °C. Control samples from mock-infected cells were prepared in the same manner.

Virus yields were assessed by titration on fully confluent cells in 96-well plates according to the method of Reed and Muench. Plates were incubated at 37 °C, and the cytopathic effect (CPE) was scored by observation under an inverted microscope.

### 3.5. Evaluation of Viral Infection

A549^ACE2/TMPRSS2^ cells were seeded in culture medium on 96-well plates (TPP, Trasadingen, Switzerland) 2 days before infection. Subconfluent cells were infected with SARS-CoV-2 viruses at 1600 50% tissue culture infectious dose (TCID50)/mL. Infection was performed in the presence of test compounds dissolved in DMSO/H_2_O or with DMSO/H_2_O as a control. After 2 h of incubation at 37 °C, cells were rinsed twice in PBS, and a fresh medium with the given inhibitor or solvent was added. The infection was carried out for 2 days, and the cytopathic effect (CPE) was assessed using the inverted light microscope. Culture supernatants were collected from all wells. The SARS-CoV-2 experiment was performed in duplicate biological replications, with individual concentrations of each compound tested in the triplicate.

### 3.6. Isolation of Nucleic Acids, Reverse Transcription, and Quantitative PCR

A viral DNA/RNA kit (A&A Biotechnology, Gdańsk, Poland) was used for nucleic acid isolation from cell culture supernatants. RNA was isolated according to the manufacturer’s instructions.

Viral RNA was quantified using quantitative PCR coupled with reverse transcription (RT-qPCR) (GoTaq Probe 1-Step RT-qPCR System, Promega, Madison, WI, USA) using a CFX96 Touch real-time PCR detection system (Bio-Rad, Munich, Germany). The reaction was carried out in the presence of the probes and primers (Fwd: CAC ATT GGC ACC CGC AAT C; Rev: GAG GAA CGA GAA GAG GCT TG; probe: 6FAM-ACT TCC TCA AGG AAC AAC ATT GCC A-BHQ-1). The heating scheme was as follows: 15 min at 45 °C and 2 min at 95 °C, followed by 40 cycles of 15 s at 95 °C and 1 min at 58 °C. In order to assess the copy number of the N gene, standards were prepared. The PCR product was amplified and cloned into pTZ57R/T plasmids using an InsTAclone PCR cloning kit (Thermo Scientific, Warszawa, Poland). The resulting plasmid was linearized, its concentration was assessed using a NanoDrop™ 2000 spectrophotometer (Thermo Fisher Scientific, Waltham, MA, USA); and the number of copies was deducted based on the Avogadro constant. The obtained standards were serially diluted and used as an input for real-time PCR.

### 3.7. Cell Viability Assay

Cell viability was evaluated using the XTT Cell Viability Assay kit (Biological Industries, Cromwell, CT, USA) according to the manufacturer’s protocol. A549^ACE2/TMPRSS2^ cells were cultured on 96-well plates. Cells were incubated with compounds for 48 h at 37 °C in an atmosphere containing 5% CO_2_. After incubation, the medium was discarded and 100 µL of fresh medium were added to each well. Then, 25 µL of the activated 2,3-bis-(2-methoxy-4-nitro-5-sulphenyl)-(2H)-tetrazolium-5-carboxanilide (XTT) solution were added, and samples were incubated for 2 h at 37 °C. The absorbance (λ = 450 nm) was measured using a Spectra MAX 250 spectrophotometer (Molecular Devices, San Jose, CA, USA). The obtained results were normalized to the control samples, where cell viability was set to 100%.

## 4. Conclusions

The results reported in this manuscript suggest that the addition of two molar equivalents of l-arginine makes rutin more soluble by two concomitant mechanisms: through an acid-base interaction and by a π-cation interaction that might hamper the formation of rutin/rutin intermolecular π-π stacking interaction observed in rutin crystal structures.

In spite of the improved solubility and of the evident Mpro inhibition ***RutinArg***, as well as Rutin, is not able to block the virus replication in a cellular model either using water or DMSO as a medium for the solubilization of the compounds. Considering that, based on docking analysis, the terminal sugar moiety does not show crucial interactions with Mpro, we suggest that the aglycon, as well as its des-rhamnosyl analogue (isoquercitrin), should be considered a good starting point for hit optimization, as demonstrated for quercetin and some of its selenium derivatives. [16,37].

## Figures and Tables

**Table 1:** Average interaction energies of ionization states i1-4 of rutin with the aqueous environment (0.15 M NaCl). R represents the rutin glycon.

Ionization State		E_int_ (kcal/mol)
i1	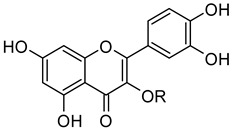	−141.15
i2	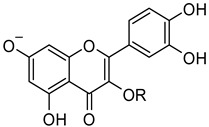	−283.72
i3	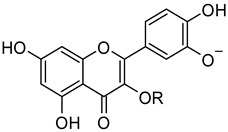	−258.74
i4	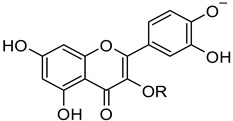	−259.89

**Table 2:** Inhibition constant (K_i_) and effective inhibition concentration 50% (IC_50_) for the compounds tested.

	Ki (µM)	IC_50_ (µM)
Quercetin (**2**) ^a^	7.4	21
Rutin (**1**)	15 ± 1	41 ± 3
l-Arginin (**3**)	ND	ND
*RutinArg*	11 ± 1	30 ± 2

^a^ taken from ref [15]; ND, not detected.

## Data Availability

All the data are within the article and the supporting information.

## Supplementary Materials

The following are available online, Figure S1: Citotoxic and antiviral evaluation; ^1^H-NMR and ^13^C-NMR of ***RutinArg*** mixture.

## Sample Availability

Samples of the compounds are available from EcoTech.
