# Supplementary material for: l-Arginine Improves Solubility and ANTI SARS-CoV-2 Mpro Activity of Rutin but Not the Antiviral Activity in Cells
Source: Molecules. 2021 Oct 7;26(19):6062. doi: 10.3390/molecules26196062 (PMC8512140; doi:10.3390/molecules26196062)
Supplement: Supplementary file 1 [file molecules-26-06062-s001.zip › molecules-1400018-supplementary.pdf]

-Supporting info-

L-Arginine improves solubility and anti SARS-CoV-2 Mpro activity of Rutin but not the antiviral activity in Cells.

Luca Sancineto[1], Carmine Ostacolo[2], David Ortega-Alarcon,[3,4] Ana Jimenez-Alesanco,[3,4] Laura Ceballos-Laita,[3,5] Sonia Vega,[3] Olga Abian,[3,4,5,6,7], Adrian Velazquez-Campoy,[3,4,6,7,8] Silvia Moretti[9], Agnieszka Dabrowska[10,11], Pawel Botwina[10,11], Aleksandra Synowiec[10], Anna Kula-Pacurar[10], Krzysztof Pyrc[10], Nunzio Iraci[12,*] and Claudio Santi[1,*]

[1] Group of Catalysis Synthesis and Organic Green Chemistry, Department of Pharmaceutical Sciences, University of Perugia, Via del Liceo 1, 06122, Perugia (PG), Italy; luca.sancineto@unipg.it
[2] Department of Pharmacy, University of Naples Federico II, 80131 Napoli, Italy; carmine.ostacolo@unina.it
[3] Institute for Biocomputation and Physics of Complex Systems (BIFI), Joint Units IQFR-CSIC-BIFI, and GBsC-CSIC-BIFI, Universidad de Zaragoza, 50018 Zaragoza, Spain; dortega@bifi.es, ajimenez@bifi.es, ceballos.laita@gmail.com, svega@bifi.es, oabifra@unizar.es; adrianvc@unizar.es
[4] Departamento de Bioquímica y Biología Molecular y Celular, Universidad de Zaragoza, 50009 Zaragoza, Spain
[5] Instituto de Investigación Sanitaria de Aragón (IIS Aragon), 50009 Zaragoza, Spain
[6] Instituto Aragonés de Ciencias de la Salud (IACS), 50009 Zaragoza, Spain.
[7] Centro de Investigación Biomédica en Red en el Área Temática de Enfermedades Hepáticas Digestivas (CIBERehd), 28029 Madrid, Spain
[8] Fundación ARAID, Gobierno de Aragón, 50018 Zaragoza, Spain
[9] Eco Tech, Ponte San Giovanni, Perugia, Italy; silvia.moretti@ecotechgroup.it
[10] Virogenetics Laboratory of Virology, Malopolska Centre of Biotechnology, Jagiellonian University, Gronostajowa 7a, 30-387 Krakow, Poland.
[11] Microbiology Department, Faculty of Biochemistry, Biophysics and Biotechnology, Jagiellonian University, Gronostajowa 7, 30-387 Krakow, Poland.
[12] Department of Chemical, Biological, Pharmaceutical, and Environmental Sciences. University of Messina. Viale Ferdinando Stagno d'Alcontres, 31, 98166 Messina, Italy;

Figure S1. Citotoxic and antiviral evaluation S2
^{1}H NMR of RutinArg S3
^{13}C NMR of RutinArg S4

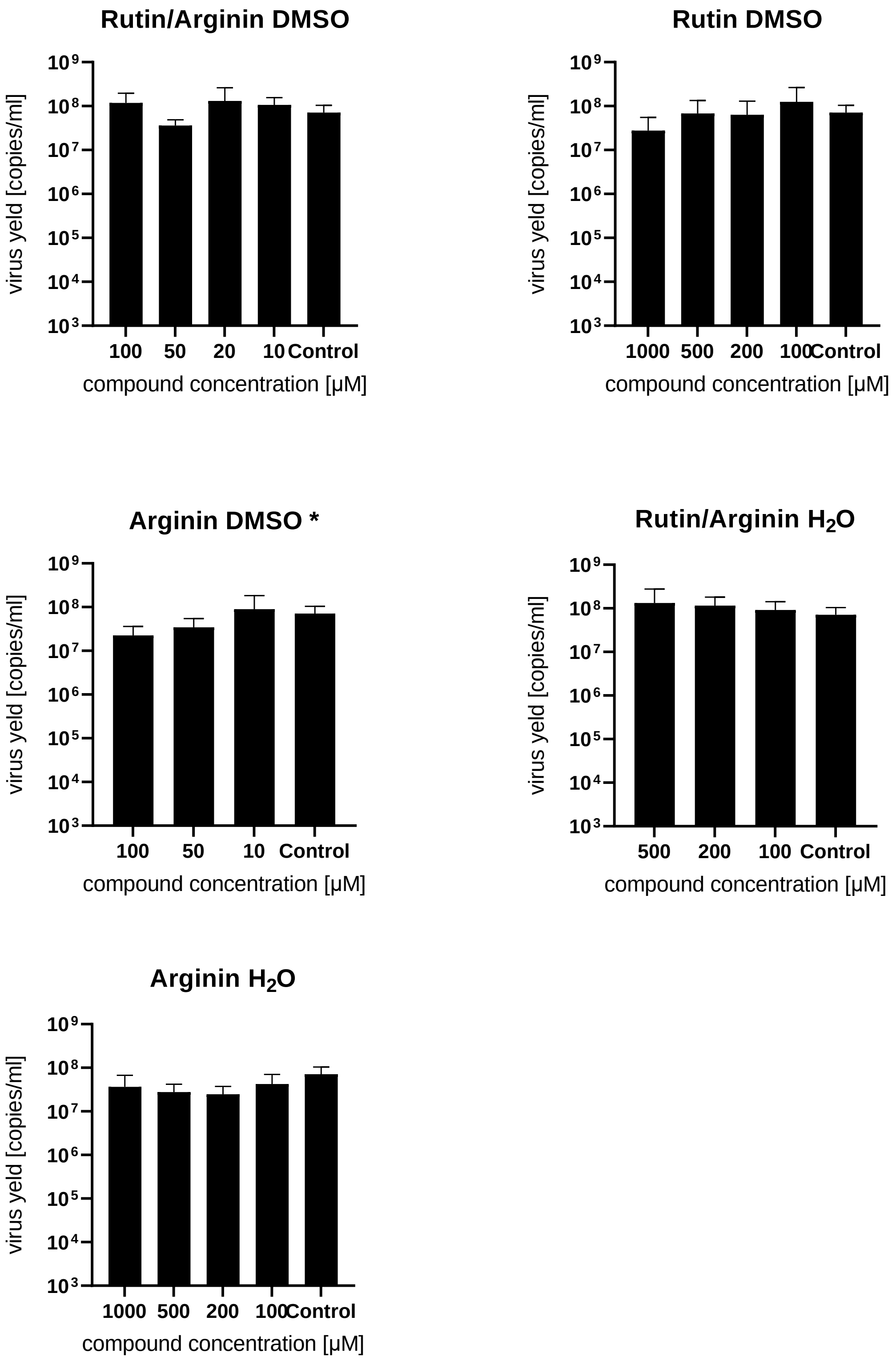

Figure S1. *Antiviral activity of rutin and its combination with arginine against SARS-CoV-2. Virus replication was evaluated using RT-qPCR. The data are presented as SARS-CoV-2 RNA copies per ml of the original sample. Bars show mean with SD. * - partial resuspension in DMSO as some precipitates were visible.*

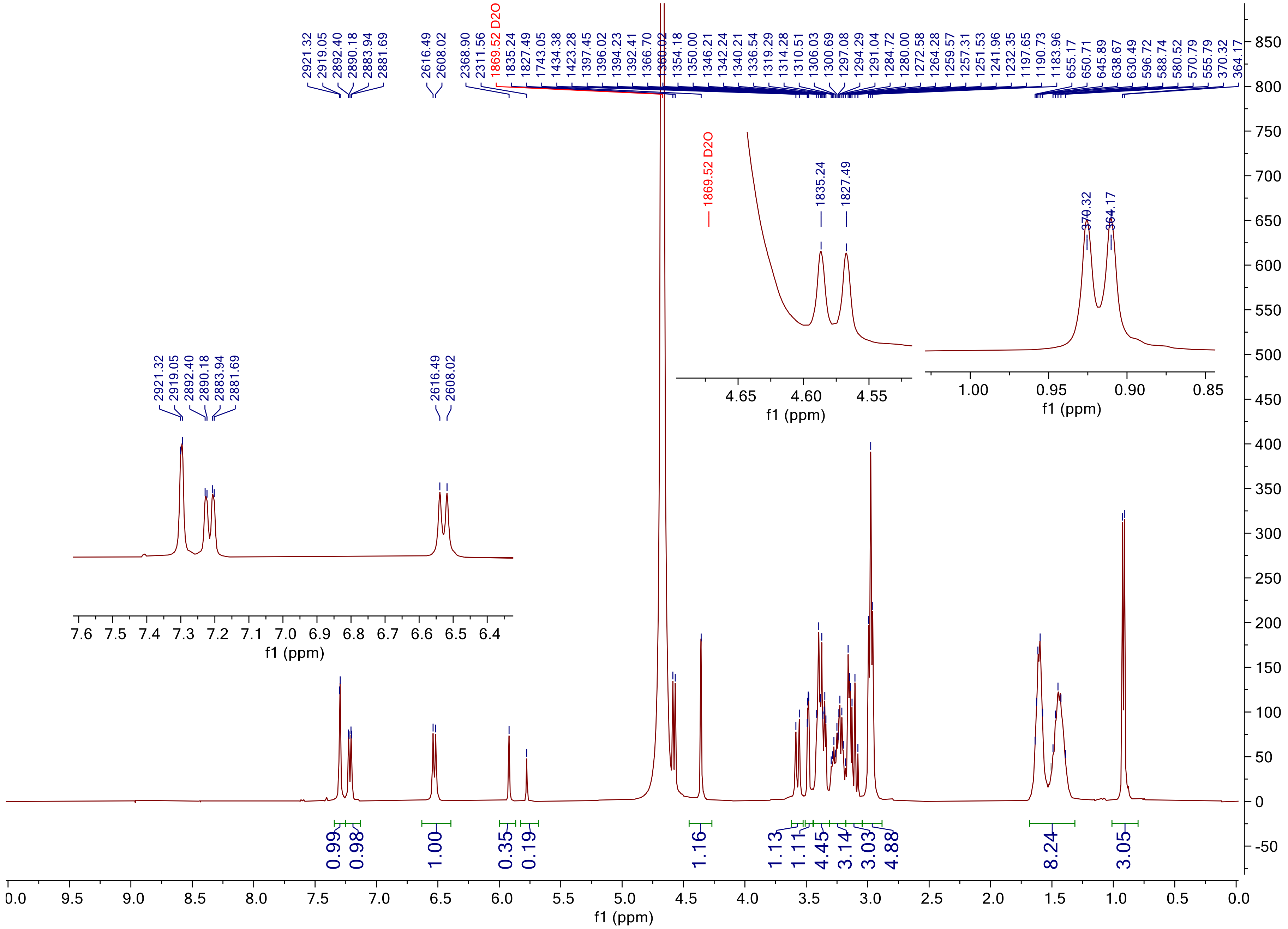

1869.52 D2O
f1 (ppm)
0.99
0.98
1.00
0.35
0.19
1.16
1.13
1.11
4.45
3.14
3.03
4.88
8.24
3.05

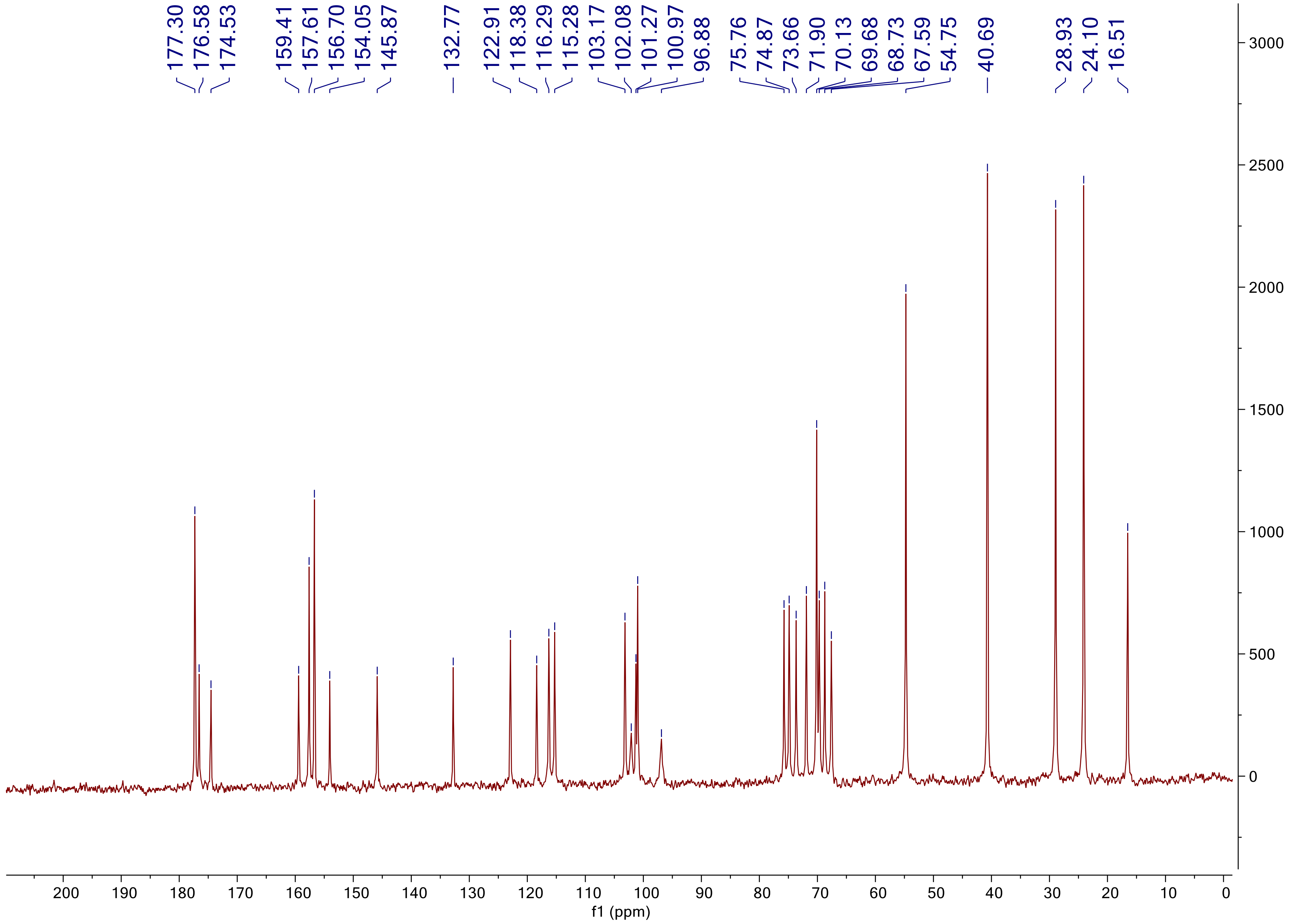

177.30
176.58
174.53
159.41
157.61
156.70
154.05
145.87
132.77
122.91
118.38
116.29
115.28
103.17
102.08
101.27
100.97
96.88
75.76
74.87
73.66
71.90
70.13
69.68
68.73
67.59
54.75
40.69
28.93
24.10
16.51
3000
2500
2000
1500
1000
500
0
200
190
180
170
160
150
140
130
120
110
100
90
80
70
60
50
40
30
20
10
0
f1 (ppm)
